# A distributional regression approach to modeling the impact of structural and intermediary social determinants on communities burdened by tuberculosis in Eastern Amazonia – Brazil

**DOI:** 10.1186/s13690-023-01147-7

**Published:** 2023-07-20

**Authors:** Clóvis Luciano Giacomet, Antônio Carlos Vieira Ramos, Heriederson Sávio Dias Moura, Thaís Zamboni Berra, Yan Mathias Alves, Felipe Mendes Delpino, Jason E. Farley, Nancy R. Reynolds, Jonas Bodini Alonso, Titilade Kehinde Ayandeyi Teibo, Ricardo Alexandre Arcêncio

**Affiliations:** 1 Interunits PhD Program in Nursing, University of São Paulo College of Nursing at Ribeirão Preto, Ribeirão Preto, Brazil; 2 Graduate Program in Public Health Nursing, University of São Paulo College of Nursing at Ribeirão Preto, Ribeirão Preto, Brazil; 3 Postgraduate Program in Nursing, Federal University of Pelotas, Pelotas, Brazil; 4 The Center for Infectious Disease and Nursing Innovation, Johns Hopkins School of Nursing, Baltimore, United States of America; 5 Research Support Center, University of São Paulo College of Nursing at Ribeirão Preto, Ribeirão Preto, Brazil; 6 Department of Maternal and Child Nursing and Public Health, University of São Paulo College of Nursing at Ribeirão Preto, Ribeirão Preto, Brazil

**Keywords:** Epidemiology, Ecological studies, Social determinants of health, GAMLSS

## Abstract

**Background:**

Tuberculosis (TB) is a disease that is influenced by social determinants of health. However, the specific structural and intermediary determinants of TB in Eastern Amazonia remain unclear. Despite being rich in natural resources, the region faces significant challenges related to poverty, inequality, and neglected diseases. The objective of this study was to use mathematical modeling to evaluate the influence of structural and intermediary determinants of health on TB in Eastern Amazonia, Brazil.

**Methods:**

This cross-sectional included all TB cases diagnosed and registered in the Notifiable Diseases Information System (SINAN) from 2001 to 2017. Data on social determinants were collected at the census tract level. The *generalized additive model for location, scale, and shape* (GAMLSS) framework was employed to identify the effect of social determinants on communities with a high TB prevalence. The Double Poisson distribution (DPO) was chosen, and inclusion of quadratic effects was tested.

**Results:**

A total of 1730 individuals were diagnosed with TB and reported in SINAN during the analyzed period. The majority were female (59.3%), aged 31 to 59 years (47.6%), identified as blacks (67.9%), and had incomplete elementary education (46.6%). The prevalence of alcoholism was 8.6% and mental illness was 0.7%. GAMLSS analyses demonstrated that the risk of community incidence of TB is associated with the proportion of the population lacking basic sanitation, as well as with the age groups of 16–31 years and > 61 years.

**Conclusions:**

The study highlights the strategic utility of GAMLSS in identifying high-risk areas for TB. Models should encompass a broader range of social determinants to inform policies aimed at reducing inequality and achieving the goals of the End TB strategy.

**Supplementary Information:**

The online version contains supplementary material available at 10.1186/s13690-023-01147-7.

**Table:** 

Text box 1. Contributions to literature
• Few studies using mathematical modeling have addressed the social and structural determinants of health.
• Many mathematical models are used for TB prediction, but they ignore differences in variance, focusing to explain the outcome.
• The literature is rich in evidence regarding the influence of social determinants of TB but lacks distinctions regarding the dimensions of these determinants.
• The determination of TB development follows a hierarchical line of causality, requiring methodological approaches that encompass this scope.
• This study contributes to the advancement of knowledge by providing evidence on the influence of structural and intermediate social determinants of TB in the Eastern Amazon.

## Background

Brazil has one of the highest burdens of tuberculosis (TB) among countries [1]. According to the latest report by the World Health Organization (WHO), Brazil reported over 66,819 cases in 2020, indicating an 18% decrease compared to the data from 2019. However, TB-related mortality has shown an increase [1].

Although Brazil has committed to ending TB by 2050, the country has faced enormous challenges in achieving this ambitious goal. This is mainly attributed to austerity measures that have reduced social benefits for lower socio-economic groups, combined with a severe economic crisis resulting from the pandemic and government policies [2]. According to forecast models, it is unlikely that the TB targets of the United Nations (UN) Sustainable Development Goals (SDGs), which aim for a 90% reduction in TB deaths by 2030, will be met [3].

There is a broad consensus that making progress in TB control will require not only investment in strengthening TB control programs, diagnostics, and treatment, but also addressing the social and structural determinants of TB. The structural factors encompass governing actions, economic and social policies, and the distribution of power, prestige, and resources based on social positions held by individuals, families, or communities [4]. Intermediary determinants comprise material circumstances, psychosocial behavior, biological factors, and the health system [4]. The literature is rich in evidence of their influence in the context of TB [4–9] but lacks distinctions regarding the dimensions of these determinants.

To conduct the present study, it was essential to establish a theoretical framework that could address knowledge gaps and guide the selection of social determinants of health. In doing so, it becomes necessary to define the term “determinant” in order to fully comprehend the intended objective. Social determinants of health are associated with the factors and mechanisms by which social conditions impact health, and they can be modified through interventions and strategic actions. These determinants encompass factors that are more closely related to individuals (microdeterminants) as well as those associated with communities, territories, or health services (macrodeterminants) [10, 11].

Another important concept that warrants discussion is health inequality, which pertains to the circumstances in which populations are situated (such as their growth, living conditions, work environments, or aging processes) and the connection between social and economic policies that impact this phenomenon. From this standpoint, the level of development within a society is characterized by its quality of life, equity in resource distribution across the social spectrum, and the extent of social protection provided by the system [10, 11]. Considering the aforementioned, the WHO Commission on Social Determinants of Health (CDSS) [12] proposed two dimensions of determinants: one that addresses the structural aspects of the State and another that pertains to intermediate determinants of health [11, 13].

For the present study, we have adopted the conceptual framework proposed by Solar and Irwin (2010) [13]. According to this framework, structural determinants encompass all the social and political mechanisms that generate, shape, and perpetuate social hierarchies, including the labor market, educational system, institutional policies, culture, and societal values. On the other hand, intermediate determinants are associated with living conditions, psychosocial factors, behavioral and/or biological aspects, and the health system itself. In order to improve health conditions, coordinated interventions that align with one another are necessary, with Primary Health Care (PHC) embodied by the Family Health Strategy serving as the axis that coordinates and articulates social protection policies, such as the *Bolsa Família* program and other related initiatives.

It is important to highlight that the determination of disease development follows a hierarchical line of causality, necessitating methodological approaches that consider this scope. Certain determinants have a closer relationship to the development of a specific disease, such as TB in the case of this study. Mathematical modeling can be valuable in exploring the contribution of health drivers to addressing the epidemic and providing evidence for End TB strategies.

However, current TB models have limitations. For instance, a systematic review conducted by Pedrazzoli et al. [4] revealed that only a few studies using mathematical modeling have addressed the underlying Social and Structural determinants, highlighting a knowledge gap. Moreover, despite the numerous mathematical models used for TB forecasting, most of them tend to overlook the differences in variance and asymmetry/imbalance, focusing primarily on means to explain the outcome [14]. This reveals a significant gap in knowledge.

Studies have shown that applying alternative regressions techniques can yield more satisfactory results. One such technique is the Generalized Additive Models for Location, Scale, and Shape (GAMLSS), which offers a broader approach by considering not only mean (or location) but also all the parameters of the conditional distribution of outcome. These parameters can be modeled as parametric or nonparametric additive functions of independent variables and/or random-effects terms [15, 16]. Therefore, the objective of this study was to use mathematical modeling to evaluate the influence of structural and intermediary determinants of health on TB in Eastern Amazonia, Brazil.

## Methods

### Study design

This is a cross-sectional study that used secondary data and mathematical modeling.

### The setting of the study

**Fig. 1:**
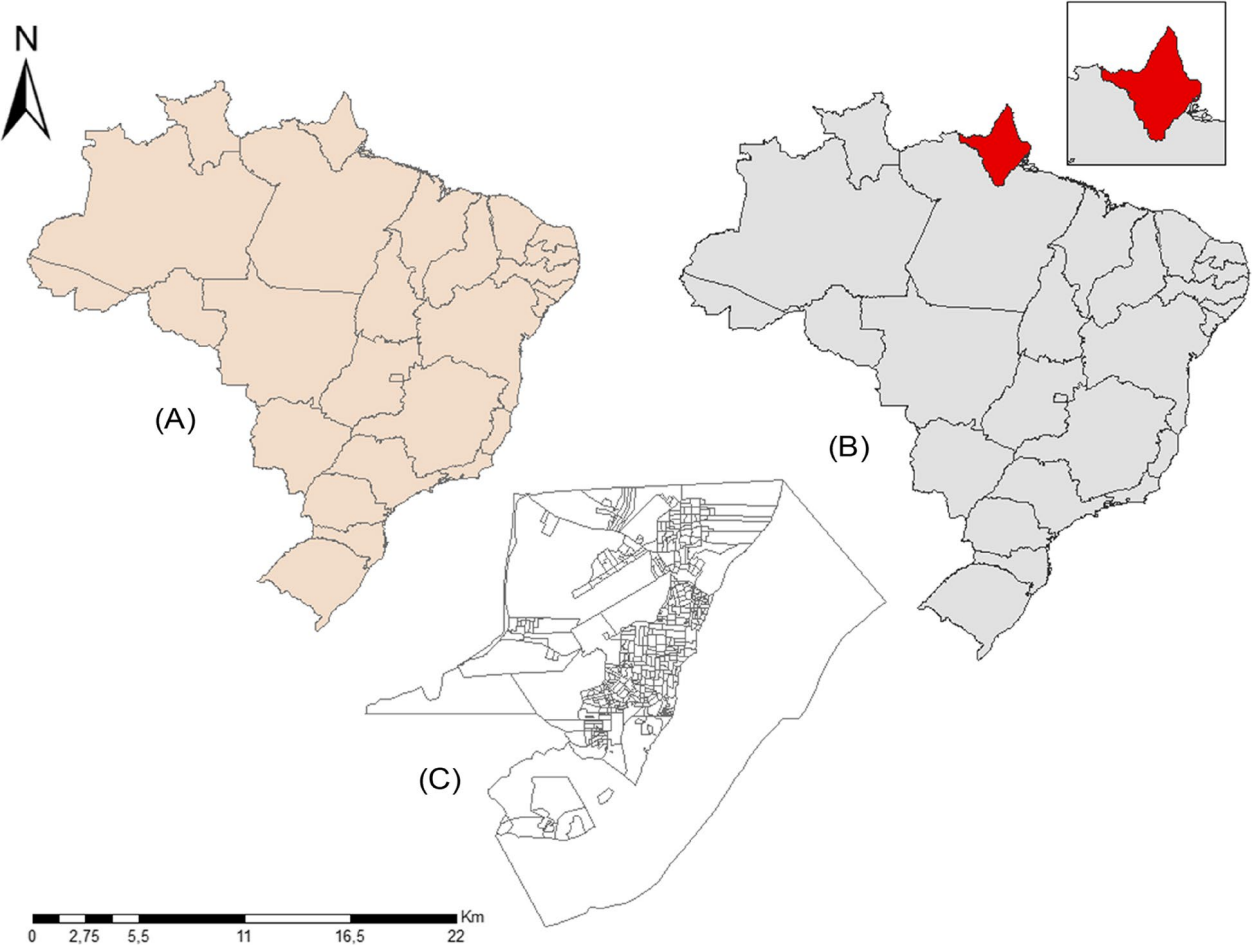
Map of the study setting

We conducted the study in Macapá, the capital of Amapá state, Eastern Amazonia (Fig. 1). It has a population of approximately 398,204 inhabitants and a demography density of around 62.14 people per km². Macapá is the most populous city in Northern Amazonas State.

Regarding TB, the city had an incidence of 17.5 cases per 100,000 in 2020, which represented a 25% decrease compared to the previous year [17]. One concerning issue is that only 39.3% of patients diagnosed with TB had confirmation through bacteriological analysis (microscopy or GeneXpert MTB/RIF), while 47.3% did not undergo a bacteriological test for confirming the TB diagnosis [18].

### The population of the study and criteria

The population comprised all cases diagnosed with TB and registered in the Notifiable Diseases Information System (SINAN) from 2001 to 2017, residing in Macapá (International Classification of Diseases (ICD) – ICD-10 A15.0 to A19.9). We collected socio-demographic data, including age, gender, race/color, education and occupation, as well as clinical information such as type of case (new or retreatment), clinical form (pulmonary or extrapulmonary), coinfection with TB-HIV (Human Immunodeficiency Virus), alcoholism, mental disorders, and comorbid TB-diabetes.

According to the WHO guidelines, the diagnosis of TB involves identifying a patient with *Mycobacterium tuberculosis complex* through a clinical specimen, which can be done through microscopy, culture, or a newer method like molecular line probe assay [19]. In Brazil, a pulmonary case with one or more initial sputum smear examinations positive for acid-fast bacilli (AFB) is also defined as a “case” [20].

In some cases, the diagnosis can be established solely through clinical examination by a physician and an X-ray; however, this method is not recommended by the Brazilian sanitary authorities. New patients are defined as individuals who have no previous history of TB treatment or who have been treated for less than one month. These patients should be prescribed a regimen consisting of six months of rifampicin: 2HRZE/4HR [20].

### Unity of study analysis and variables

The georeferencing of TB cases reported in Macapá was initially conducted by retrieving the geographic coordinates (latitude and longitude) of the residential addresses obtained from the SINAN notification form using the freely accessible software Google Earth. Subsequently, georeferencing was performed by assigning a geographical representation to each address record, creating a shapefile of points using ArcGIS software version 10.5.

The unit of analysis for the study was the 811 Urban Census Tract (UCT) within Macapá, obtained from the Brazilian Institute of Geography and Statistics (IBGE). UCTs are the smallest territorial units in an urban area, characterized by a defined size, number of households, and number of residents. They are commonly used for Brazilian surveys and statistical research [13]. By combining the georeferenced cases shapefile with the UCT shapefile obtained from IBGE, we were able to determine the corresponding UCT for each case.

**Fig. 2:**
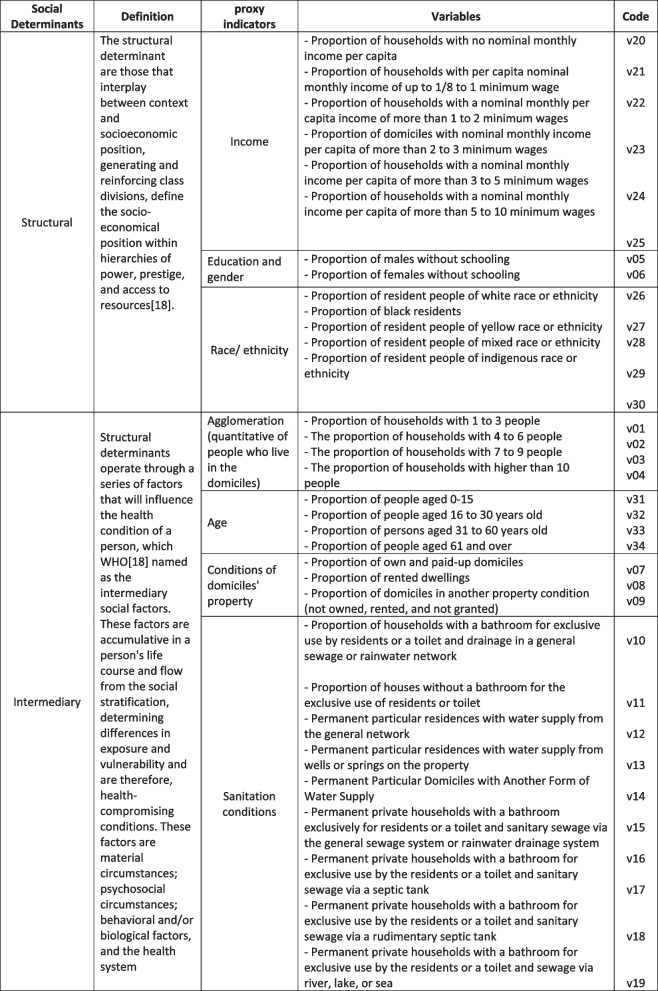
Structural and intermediary determinants selected for the study, Eastern Amazonia, Brazil

The information regarding UCT was obtained from the 2010 Brazilian Demographic Census, which collected data on both structural and intermediary determinants such as household conditions and characteristics of the territories (UCTs). Figure 2 shows the variables under study.

### Analysis plan

Initially, the variables were analyzed using descriptive statistics. Additionally, to identify the structural and intermediary determinants associated with TB, we employed the GAMLSS model [15]. This model was chosen because the response variable (TB cases) did not follow a distribution from the exponential family, and exhibited heterogeneity in terms of distribution scale and shape. The response variable was influenced by the explanatory variables [21].

Let yT = (y1, …, yn) be a vector of size n representing the response variable with a density function f(yi | i), where i = 1, 2, …, n. Let k = 1, 2, 3, 4, and let gk(.) be a monotone link function that relates the parameters to the independent variables based on the following equations:

Since ${y}^{T}=({y}_{1}, \dots , {y}_{n})$ is a vector of size n of the response variable with density function $f\left({y}_{i}\right|{\theta }^{i}$, where${\theta }^{i}=\left({\theta }_{1i},{\theta }_{2i} {\theta }_{3i} {\theta }_{4i}\right)=({\mu }_{i},{\sigma }_{i} {\nu }_{i} {\tau }_{i})$, and let k = 1,2,3,4 e let${g}_{k}(.)$ a monotone link function that relates the parameters to the independent variables based on the following equations:

$$\{{g}_{1}\left(\mu \right)={n}_{1}={X}_{1}{\beta }_{1}+{\varSigma }_{j=1}^{{J}_{1}}{Z}_{j1}{\gamma }_{j1}, {g}_{2} \left(\sigma \right)={n}_{2}={X}_{2}{\beta }_{2}+\sum\limits_{j=1}^{{J}_{2}}{Z}_{j2}{\gamma }_{j2,} {g}_{3}\left(\nu \right)={n}_{3}={X}_{3}{\beta }_{3}+{\varSigma }_{j=1}^{{j}_{3}}{Z}_{{j}_{3}}{\gamma }_{{j}_{3},} {g}_{4}\left(\tau \right)={n}_{4}={X}_{4}{\beta }_{4}+{\varSigma }_{j=1}^{{J}_{4}}{Z}_{j4}{\gamma }_{j4.}$$

Where µ, σ, ν e τ are vectors of length n, ${\beta }_{k}=\left({\beta }_{1k},{\beta }_{2k}, \dots , {\beta }_{{j}^{k}}\right)$ is a vector of length${j}^{k}$ e${X}_{k}$ is the delineation matrix of order n x ${j}^{k}$. The function${h}_{jk}$

Non additive function of the independent variable ${X}_{k}$ evaluated at ${x}_{jk}$.

The selection of the distribution for the dependent variable was conducted using the Generalized Akaike Information Criterion (GAIC), defined by $GAIC= -2 L\left(\widehat{\theta }\right)+bdf$, where $l\left(\widehat{\theta }\right)$ represents the likelihood function, b is a penalty parameter, and df denotes the degrees of freedom of the model [22]. For b = 2 we have the original Akaike Information Criterion (AIC). As stated in the literature [23], all distributions falling within the GAMLSS class were considered.

The selection of independent variables was carried out in two steps. In the first step, the presence of multicollinearity among the independent variables was assessed. Multicollinearity evaluation determines the inclusion of variables in the model that are highly correlated with each other. One commonly used measures is the Variance Inflation Factor (VIF), whose expression is defined by:

$${VIF}_{j}= \frac{1}{1- {R}_{j}^{2}}$$

Where ${R}_{j}^{2}$ is the multiple correlation coefficient resulting from regressing${ X}_{j}$ on the other p – 1 regressor. The higher the degree of dependence of${ X}_{j}$ on the remaining regressors, the stronger the dependence and the higher the value of ${R}_{j}^{2}$. A VIF value greater than 5 was chosen as the cutoff point [16, 24]. The VIF values can be found in the additional file with more details (see Additional file 1).

In addition, we applied the stepwise method using the Generalized Akaike Information Criterion with k = 4 [12] for the remaining variables from the first stage. Following this analysis, the Double Poisson Distribution (DPO) was selected based on the AIC value (Table 3). The DPO (µ, σ) has the following probability density function [23].

$$f\left(y\right|\mu ,\sigma )=(\frac{1}{\sigma }{)}^{\frac{1}{2}} {e}^{-\frac{\mu }{\sigma }}\left(\frac{{e}^{-y}{y}^{y}}{y!}\right)(\frac{e\mu }{y}{)}^{y/\sigma }C$$

Where y = 0, 1, 2, …, ∞, µ > 0 and $\sigma$ > 0, where C is a proportionality constant that is calculated numerically. The link function between the parameters and the independent variables is the logarithmic function, i.e., g_1 (*µ*) = log(µ) and g_2 ($\sigma$) = log(σ) [23].

We assessed the adequacy of the model through diagnostic graphics, including Fitted Values x Residuals, Order of Observations x Residuals, Distribution of Residuals, and Quantile-Quantile plot (Q-Q plot). Additionally, the Shapiro-Wilk Normality test was applied to the model’s residuals to verify if they fit the Standard Normal distribution. The dependent variable, the number of TB cases in each UCT, and the indicators of structural and intermediary determinants were considered as independent variables (Table 1). These variable/proxy indicators were selected based on the theoretical framework defined in the study [4, 21].

**Table 1:** Socio-demographic and clinical-epidemiological profile of Tuberculosis cases, Eastern Amazonia, Brazil

Variables	*N* = 1,730 (%)
**Age (years)**	
0 to 14 years	74 (4.3)
15 to 30 years old	640 (37.0)
31 to 59 years	823 (47.6)
Above 60 years old	175 (10.1)
Blank/Ignored	18 (1.0)
**Gender**	
Male	704 (40,7)
Female	1026 (59,3)
**Education**	
Illiterate	128 (7.4)
Elementary school complete	112 (6.5)
Elementary School Incomplete	803 (46.6)
High School Complete	224 (12.9)
Higher Education Incomplete	52 (3.0)
Higher education complete	127 (7.3)
Blank/Ignored	284 (16.4)
**Registration types**	
New	682 (39.4)
Relapse	9 (0.5)
Retreatment after abandonment	0 (0.0)
Retreatment	1 (0.1)
Transfer	1 (0.1)
After death	26 (1.5)
Blank/Unknown	1.011 (58.4)
**Clinic form**	
Extrapulmonary	236 (13.6)
Pulmonary	1472 (85.1)
Pulmonary + Extrapulmonary	18 (1.0)
Blank/Ignored	4 (0.3)
**Coinfection TB-HIV**	
No	787 (45.5)
Yes	77 (4.5)
Blank/Ignored	866 (50.0)
**Coinfection TB-Diabetes**	
No	912 (52.8)
Yes	94 (5.4)
Blank/Ignored	724 (41,8)
**Alcoholism**	
No	896 (51.7)
Yes	148 (8.6)
Blank/ Ignored	686 (39.7)
**Mental Disorders**	
No	1001 (57.8)
Yes	12 (0.7)
Blank/Ignored	717 (41.5)

We performed GAIC selection of the independent variables considering only the linear effects. Additionally, we tested the inclusion of quadratic effects since the scatter plots suggested a possible quadratic relationship based on the fitted curve (by the loess method = local polynomial regression). To compare the two models (linear terms versus quadratic terms), we used the Likelihood Ratio Test (LRT). Once we selected the best-fitting model, we estimated the Relative Increase, expressed in percentage, in the Average Number of Tuberculosis Cases through the expression $AR\left(\beta \right)=\left(\beta \right) - 1]*100\%$. The data analysis for this part was conducted using R program version 4.1.1 with the GAMLSS library [25].

## Results

A total of 1,730 people were diagnosed with TB and reported in SINAN between 2001 and 2017. Table 1 shows the main characteristics of the patients, revealing an age range of 1 to 89 years (median = 44.5 years). The majority were female (59.3%), between the ages of 31 to 59 years (47.6%), identified as black (67.9%), and had incomplete elementary school education (46.6%). In terms of the clinical and epidemiological profile, most cases were classified as new (39.4%) and pulmonary (85.1%) TB. Regarding comorbidities, there was a prevalence of 4.5% for TB-HIV coinfection, 5.4% for TB-diabetes, 8.6% for alcoholism, and 0.7% for mental illness. It is important to note that there was an excess of cases with missing or blank data.

**Fig. 3:**
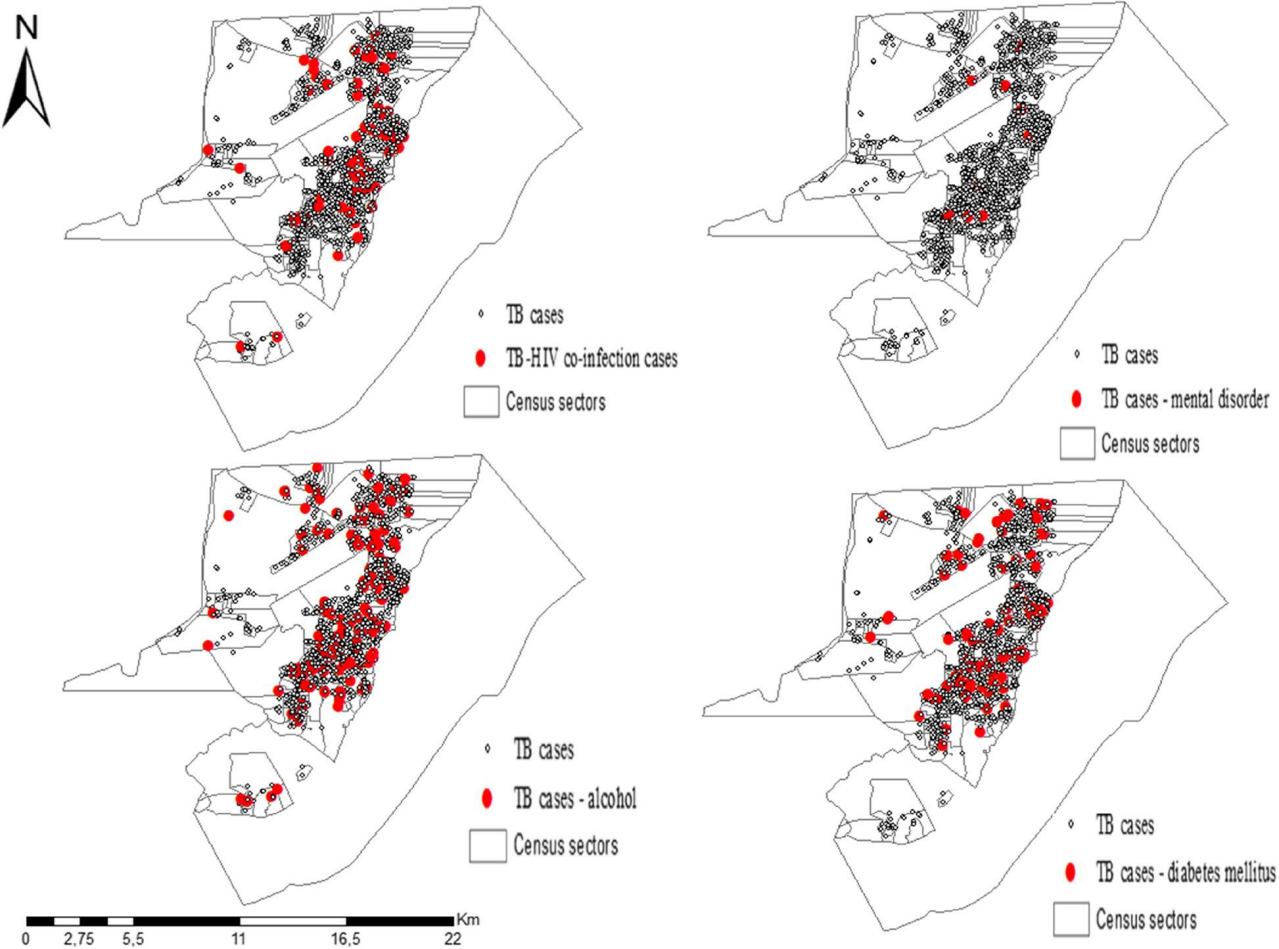
Spatial distribution of tuberculosis in the study, Eastern Amazonia, Brazil

Figure 3 displays the spatial distribution of TB cases, considering the comorbidities or health conditions: TB-HIV coinfection, mental disorder, diabetes or alcoholism.

In Table 2, we presented the key information derived from the descriptive statistics. The dependent variable includes the total number of cases, while the independent variables (proxy variables of structural and intermediary social determinants) represent the proportion of households in each UCT that exhibit specific conditions as indicated by the respective variable.

**Table 2:** Statistics descriptive of the variables defined in the study, Eastern Amazonia, Brazil

Variables	Mean	Standard Deviation	Minimum	Q0.25	Q0.5	Q0.75	Maximum
Cases of TB per Urban Census Tract	3.43	3.00	0.00	1.00	3.00	5.00	23.00
v1 Proportion of households with 1 to 3 people	42.32	7.56	19.15	37.77	42.21	47.19	76.81
v2 Proportion of households with 4 to 6 people	43.79	5.42	15.94	40.70	43.81	47.34	56.52
v3 Proportion of households with 7 to 9 people	10.82	4.07	0.00	8.28	10.17	12.86	29.79
v4 Proportion of households with higher than 10 people	3.08	2.19	0.00	1.60	2.70	4.04	14.89
v5 Proportion of males without schooling	18.33	7.28	3.28	13.09	17.60	22.73	44.06
v6 Proportion of females without schooling	17.14	6.82	2.33	12.33	16.12	21.38	43.90
v7 Proportion of own and paid-up domiciles	76.05	12.20	17.55	69.28	77.94	84.40	100.00
v8 Proportion of rented dwellings	16.70	9.72	0.00	9.45	15.32	22.65	67.11
v9 Proportion of domiciles in another property’ condition (not owned, rented, and not granted)	0.19	0.67	0.00	0.00	0.00	0.00	10.20
v10 Proportion of households with a bathroom for exclusive use by residents or a toilet and drainage in a general sewage or rainwater network	65.63	14.40	9.09	56.64	65.60	75.00	100.00
v11 Proportion of houses without a bathroom for the exclusive use of residents or toilet	1.11	1.86	0.00	0.00	0.36	1.48	12.00
v12 Permanent particular residences with water supply from the general network	59.39	36.91	0.00	22.02	70.27	94.16	100.00
v13 Permanent particular residences with water supply from wells or springs on the property	36.73	34.79	0.00	4.49	25.65	70.83	100.00
v14 Permanent Particular Domiciles with Another Form of Water Supply	3.28	7.78	0.00	0.00	0.80	2.88	82.50
v15 Permanent private households with a bathroom exclusively for residents or sanitary	98.29	4.36	22.33	97.89	99.32	100.00	100.00
v16 Permanent private households with exclusive use of bathroom or toilet and sanitary sewage via the general sewage network or rainwater drainage	10.06	21.83	0.00	0.00	1.06	5.27	98.90
v17 Permanent private households with a bathroom for exclusive use by the residents or a toilet and sanitary sewage via a septic tank	18.73	26.59	0.00	0.88	6.02	25.09	100.00
v18 Permanent private households with a bathroom for exclusive use by the residents or a toilet and sanitary sewage via a rudimentary septic tank	49.23	33.94	0.00	14.18	54.88	81.91	99.36
v19 Permanent private households with a bathroom for exclusive use by the residents or a toilet and sewage via river, lake, or sea	17.28	28.16	0.00	0.00	1.09	21.06	100.00
v20 Proportion of households with no nominal monthly income per capita	4.07	4.83	0.00	1.31	2.68	5.06	38.64
v21 Proportion of households with per capita nominal monthly income of up to 1/8 to 1 minimum wage	58.02	20.22	7.63	43.61	60.00	73.71	96.09
v22 Proportion of households with a nominal monthly per capita income of more than 1 to 2 minimum wages	18.82	7.02	2.61	13.28	20.19	24.24	38.94
v23 Proportion of domiciles with nominal monthly income per capita of more than 2 to 3 minimum wages	7.75	5.63	0.00	3.14	6.85	11.41	36.36
v24 Proportion of households with a nominal monthly income per capita of more than 3 to 5 minimum wages	6.47	5.70	0.00	1.64	5.00	10.21	24.29
v25 Proportion of households with a nominal monthly income per capita of more than 5 to 10 minimum wages	3.93	4.57	0.00	0.71	2.24	5.55	23.25
v26 Proportion of households with nominal monthly income per capita higher than 10 minimum wages	1.20	2.05	0.00	0.00	0.42	1.42	
v27 Proportion of resident people of white race or ethnicity	27.10	6.51	5.88	23.31	26.96	30.70	14.12
v28 Proportion of black residents	9.18	5.49	0.00	5.63	8.57	11.57	52.63
v29 Proportion of resident people of yellow race or ethnicity	1.19	1.34	0.00	0.26	0.80	1.80	39.60
v30 Proportion of resident people of mixed race or ethnicity	62.34	8.35	31.15	57.59	61.96	67.50	94.12
v31 Proportion of resident people of indigenous race or ethnicity	0.20	0.51	0.00	0.00	0.00	0.16	
v32 Proportion of people aged 0–15	32.74	7.21	9.44	27.46	32.64	38.28	6.77
v33 Proportion of people aged 16 to 30 years old	31.35	3.12	23.23	29.43	31.16	33.26	51.47
v34 Proportion of persons aged 31 to 60 years old	32.76	5.08	19.22	29.36	32.72	36.37	54.44
v35 Proportion of people aged 61 and over	5.16	2.93	0.87	3.21	4.16	6.10	47.73

Table 3 displays the outcomes obtained from applying the AIC criterion to select the most suitable probability distribution for the total number of TB cases. It reveals that the DPO distribution yielded the best result, as it obtained the highest AIC value.

**Table 3:** The main distributions for fitting a GAMLSS model selected in accordance with the Akaike Information Criterion value, Eastern Amazonia, Brazil

Distribution for fitting a GAMLSS model	Akaike information criterion (AIC)
The Double Poisson distribution (DPO)	2004.25
Zero Inflated Negative Binomial Distribution (ZINBI)	2006.04
Zero adjusted negative binomial distribution (ZANBI)	2006.04
Zero adjusted (hurdle) of the beta negative binomial distribution (ZABNB)	2008.02
Zero-inflated of the beta negative binomial distribution (ZIBNB)	2008.02
The Zipf and zero adjusted Zipf distributions (ZINBF)	2008.04
The Sichel distribution (ZISICHEL)	2008.04
Zero-inflated Poisson inverse Gaussian distribution (ZIPIG)	2008.22
Negative Binomial type II distribution (SNBII)	2010.27
Negative Binomial type I distribution (SBNBI)	2010.27

**Fig. 4:**
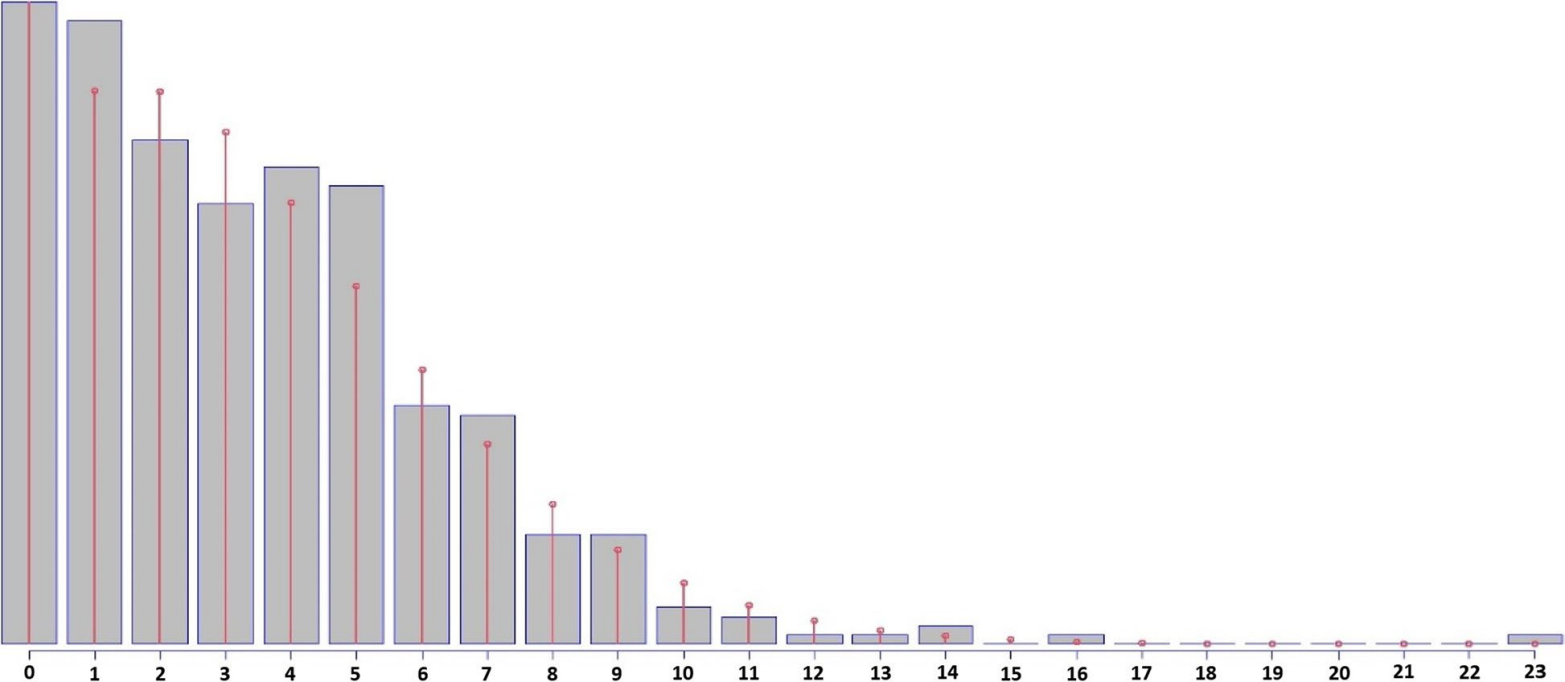
Histograms of Tuberculosis cases with fit to the transformed Double Poisson distribution, Eastern Amazonia, Brazil

In Fig. 4, we present the overlay of the DPO density on the data distribution. The application of the Shapiro-Wilk normality test on the model’s residuals indicates that the fit is appropriate (W= 0.9967; *p*-value = 0.5245).

Table 4 includes the statistics obtained from the modeling analysis. It showcases the complete model (saturated) selected through GAIC without outlier, incorporating quadratic terms while excluding quadratic outlier (Proportion of people aged 16 to 30 years old (v33) and Permanent private households with a bathroom for exclusive use by the residents or a toilet and sewage via river, lake, or sea (v19)). After conducting the LR test, the model with quadratic terms exhibited better fit than the linear model. In Table 4, v19^1 represents the linear term of Permanent private households with a bathroom for exclusive use by the residents or a toilet and sewage via river, lake, or sea (v19) and v19^2 represents the quadratic term of the same variable. The same explanation applies to the Proportion of people aged 16 to 30 years old (v33).

**Table 4:** Model of the structural and intermediary social determinants associated with community TB infection in Eastern Amazonia, Brazil (*n* = 1,730 TB cases)

**µ**	**Estimate**	**Std. Error**	**t value**	**Pr(>|t|)**
(Intercept)	0.4331	0.2063	2.0995	0.0364*
v19^1 Permanent private households with a bathroom for exclusive use by the residents or a toilet and sewage via river, lake, or sea	-6.9247	1.9192	-3.6081	0.0003*
v19^2 Permanent private households with a bathroom for exclusive use by the residents or a toilet and sewage via river, lake, or sea	-3.4713	1.1011	-3.1526	0.0017*
v33^1 Proportion of people aged 16 to 30 years old	2.6266	0.9350	2.8091	0.0052*
v33^2 Proportion of people aged 16 to 30 years old	-2.4703	1.0115	-2.4423	0.0150*
v17 Permanent private households with a bathroom for exclusive use by the residents or a toilet and sanitary sewage via a septic tank	0.0044	0.0023	1.9035	0.0577
v35 Proportion of people aged 61 and over	0.0731	0.0163	4.4791	0.0000*
v18 Permanent private households with a bathroom for exclusive use by the residents or a toilet and sanitary sewage via a rudimentary septic tank	0.0042	0.0021	2.0369	0.0423*
**Σ**	**Estimate**	**Std. Error**	**t value**	**Pr(>|t|)**
(Intercept)	0,7195	0,0761	9,4514	0,0000*

Shapiro-Wilk normality test: 0.99681, *p*-value: 0.5654

Likelihood Ratio Test for nested GAMLSS models;

(No check whether the models are nested is performed). Null model: deviance = 1884.811 with 7 deg. of freedom

Alternative model: deviance = 1866.812 with 9 deg. of freedom; LRT = 17.99909 with 2 deg. of freedom and *p*-value = 0.0001234661

*p < 0.05

Based on the mathematical model, we identified an association between territories with a high number of tuberculosis cases and the absence or deficiency of a sewage disposal system. Additionally, there is a correlation between the prevalence of younger individuals (16 to 30 years old) or older individuals (over 61 years old) in these territories.

**Fig. 5:**
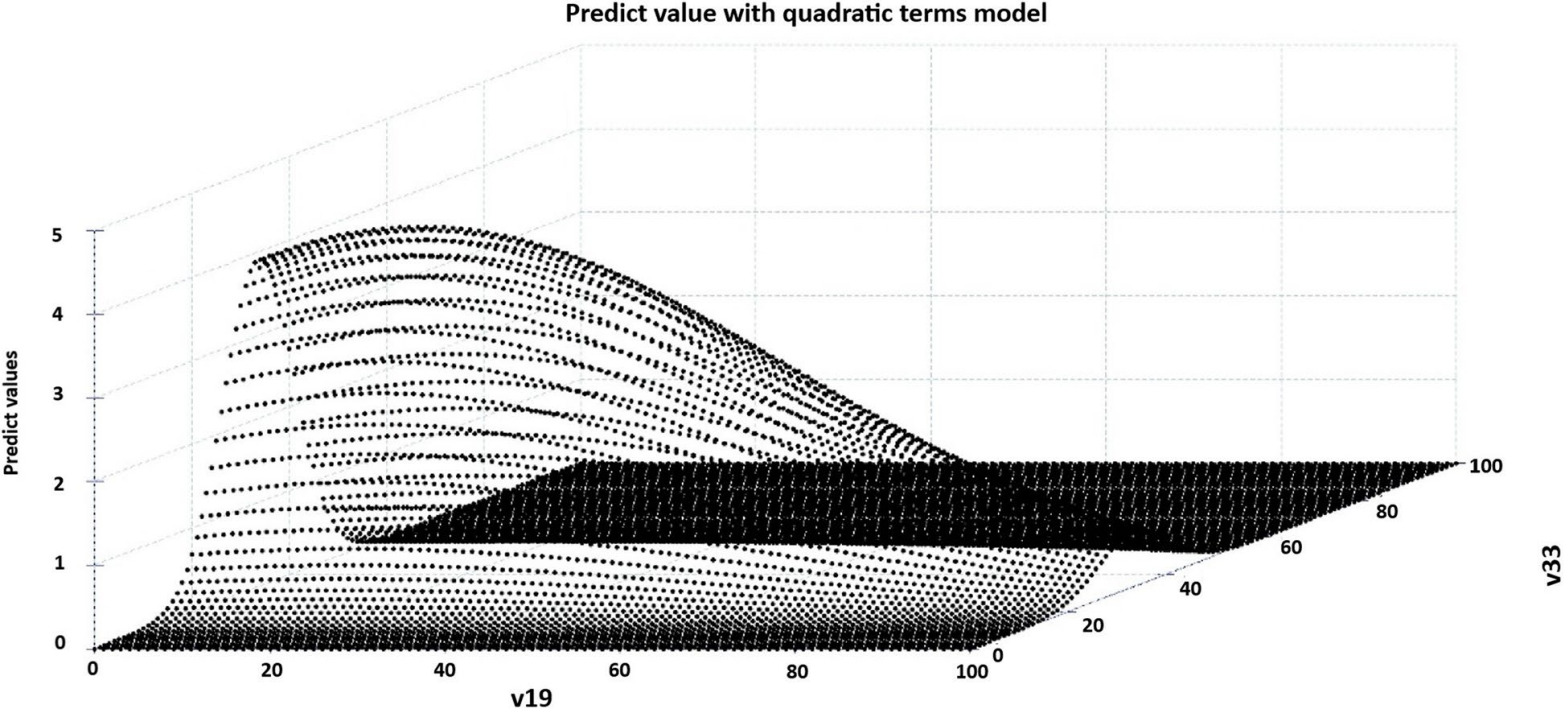
Graph with predicted values obtained from the modeling with quadratic terms, Eastern Amazonia, Brazil

In Fig. 5, we present the graph of predicted values, which includes the variables with quadratic terms: Permanent private households with a bathroom for exclusive use by the residents or a toilet and sewage via river, lake, or sea (v19) and Proportion of people aged 16 to 30 years old (v33). Regarding the variables Permanent private households with a bathroom for exclusive use by the residents or a toilet and sanitary sewage via a septic tank (v17), Permanent private households with a bathroom for exclusive use by the residents or a toilet and sanitary sewage via a rudimentary septic tank (v18) and Proportion of people aged 61 and over (v35), they have identical medians. In other words, Permanent private households with a bathroom for exclusive use by the residents or a toilet and sanitary sewage via a septic tank (v17) = 18.74%, Permanent private households with a bathroom for exclusive use by the residents or a toilet and sanitary sewage via a rudimentary septic tank (v18) = 49.35% and Proportion of people aged 61 and over (v35) = 5.17%.

**Fig. 6:**
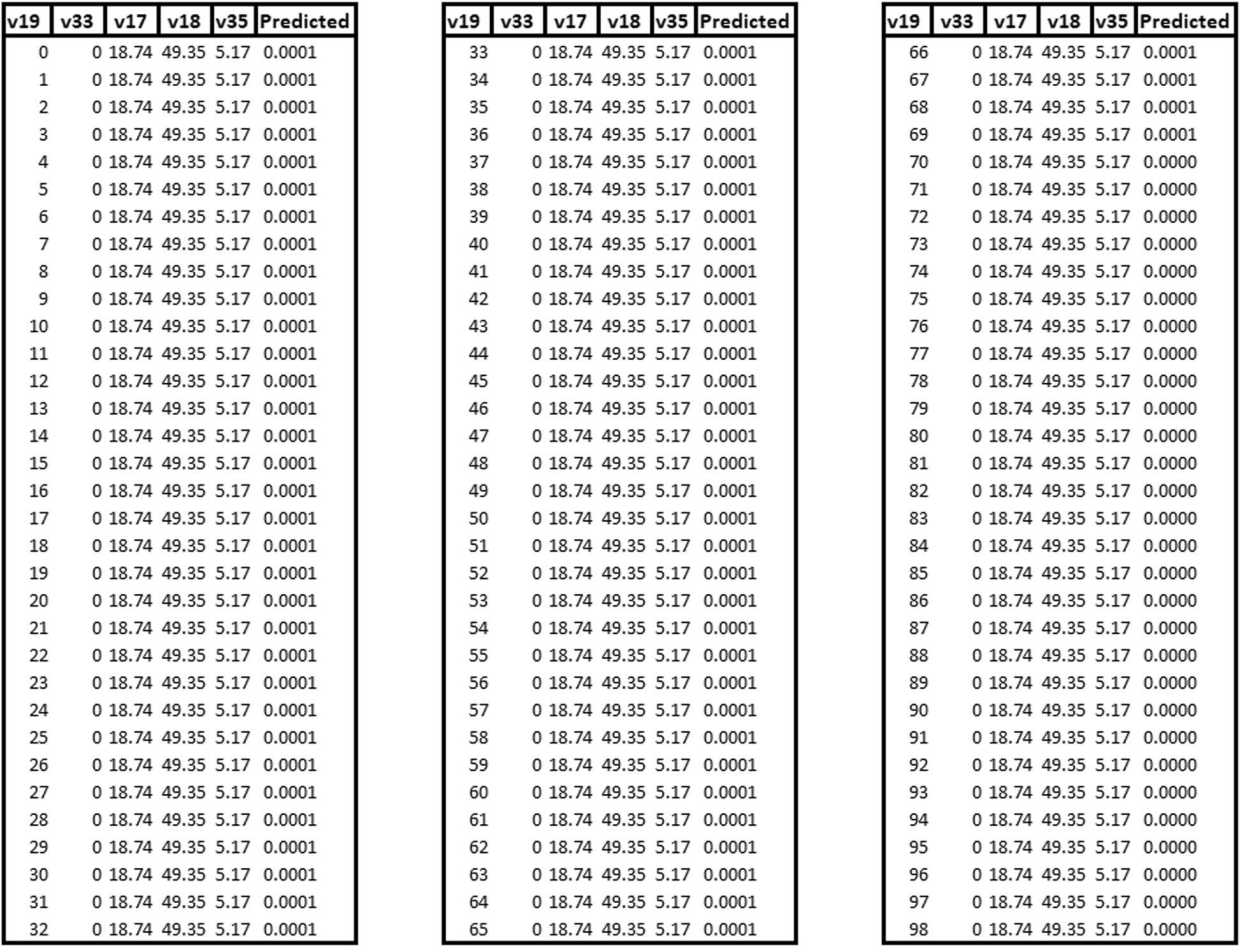
Predicting the number of cases according to the range value of Permanent private households with a bathroom for exclusive use by the residents or a toilet and sewage via river, lake, or sea (v19) and Proportion of people aged 16 to 30 years old (v33)

In this case, if we consider that v19 = 10% and v33 = 20%, the expected average number of TB cases is 0.7367. When we consider Permanent private households with a bathroom for exclusive use by the residents or a toilet and sewage via river, lake, or sea (v19) = 20% and Proportion of people aged 16 to 30 years old (v33) = 30%, the expected average number of cases is 3.7824. And if we consider Permanent private households with a bathroom for exclusive use by the residents or a toilet and sewage via river, lake, or sea (v19) = 30% and Proportion of people aged 16 to 30 years old (v33) = 40%, the average expected number of cases is 2.86. Figure 6 depicts the number of cases according to the range of values for permanent private households with either a toilet exclusively for residents’ use or a toilet connected to a sewer via a river, lake, or sea (v19), as well as the proportion of individuals aged 16 to 30 years (v33). The data complete utilized for this prediction can be found in the additional file with more details (see Additional file 2).

## Discussion

We have found evidence that GAMLSS is superior when compared to other techniques used to study social determinants [16]. The first advantage is that GAMLSS estimates the complete conditional distribution, enabling better estimation of the response variable using probability distributions such as gamma or lognormal distribution [26]. Another advantage is that all necessary resources are available through R packages, allowing the adjustment of over 50 different distribution types.

Several studies [6, 27–30] have already shown that TB and vulnerability to the disease are strongly influenced by social factors. Thus, this research provides a reflection on how TB can be influenced by the social determinants of health from a mathematical perspective through the construction of models.

It is worth noting that, according to the WHO, the social determinants of health involve non-medical factors that influence health outcomes, including the conditions in which people are born, grow, work, live, and age. It also encompasses a broader set of forces and systems that shape daily living conditions, such as the organization of healthcare systems and public policies, as well as economic systems, development agendas, social norms, social policies, and political systems in general [31, 32].

Through our findings, we identified the structural determinants (represented by age) and intermediaries (expressed by sanitary conditions of the environment, such as treated sewage). The literature has provided evidence that males of economically active age were more commonly affected by TB, which could be related to our findings [33].

According to the national data, nearly 8.5% of TB cases diagnosed in 2017 were in individuals aged 19 or younger [33]. In Brazil, TB in adolescents has long been a hidden pandemic and continues to be neglected. Based on the mathematical model presented in the present study, it was possible to find an association between the territories with the highest number of tuberculosis cases and the prevalence of younger people (16 to 30 years old). Thus, TB in adolescents needs to be considered a sentinel event since it is related to a recent infection through contact with a bacilliferous adult [33].

Another issue that may be associated with our findings is a situation revealed by a recent survey conducted by IBGE, which concluded that the country has 14.8 million unemployed people, representing 14.7% of the economically active population. However, this rate is even higher among young people. In the age group 14 to 17, 46% are actively seeking employment, and among those aged 18 to 24, unemployment affects 31% of individuals, placing them in an extremely socially vulnerable situation. The study also revealed that long-term unemployment is predominantly observed among individuals between the ages of 17 and 29. Although this data is specific to Brazil, when considering data from Eastern Amazonia, this situation can become even more serious [34], since, according to the IBGE, the North region of Brazil is the second region with the highest unemployment rates in Brazil, behind only the Southeast region [35].

It is also worth noting that in these regions, economic segregation tends to be visibly higher, mainly due to high population density and a concentration of poverty, factors that increase the risk of TB [36]. Thus, TB is still considered today a serious global public health problem directly associated with social issues.

Therefore, income and social protection, education, unemployment and job insecurity, working and living conditions, food insecurity, housing, basic amenities, and environment, early childhood development, social inclusion and non-discrimination, structural conflict, access to affordable and decent quality health services are examples of social determinants of health that can positively and/or negatively influence health equity [31].

We also found an association with territories characterized by a predominance of elderly people (age older than 61 years old). The literature has demonstrated that the elderly are more susceptible to falling into poverty compared to other age groups [37]. This vulnerability arises because the elderly have a lower probability of recovering from a negative income shock and encounter difficulties in (re)entering the labor market due to declining productivity and employability after approximately the age of 60. Consequently, poverty tends to become a more enduring condition among the elderly compared to other segments of society [37], rendering them vulnerable to both poverty and TB determinants [38].

A study conducted in Eastern Amazonia has revealed an increase in TB cases among the elderly population. This rise can be attributed to the deterioration of social conditions resulting from austerity policies implemented in Brazil. These policies have particularly weakened social security systems, which are responsible for providing social protection to the elderly. As a consequence, the elderly face challenges in accessing nutritious food, maintaining a good quality of life and improving their housing conditions [39].

We also observed that the conditions conducive to the spread of TB are associated with access to basic sanitation. This represents another significant issue observed in developing countries. According to the literature, over 2 billion people worldwide, which accounts for more than 25% of the global population, do not have access to basic sanitation [40]. A study evaluating access to basic sanitation in Brazil revealed that nearly of the 90% of the residents in Macapá lack access to the sewage network, and invest less than 30% of their financial resources picked up with taxes [41], as in the whole country.

Investments in improving housing conditions and basic sanitation are essential for enhancing people’s quality of life and preventing neglected diseases such as TB. The Sustainable Development Goals, also known as the Global Goals, aim to harmonize economic growth, environmental sustainability, and social advancement. Their objective is to ensure equal opportunities for all individuals, enabling them to lead better lives without compromising the planet’s well-being [42]. One of these goals is to achieve universal and equitable access to basic sanitation and clean water by 2030. However, attaining these goals poses a challenge in Brazil, particularly in Eastern Amazonia.

Eastern Amazonia has the largest percentage of its territory designated as legally protected areas for natural conservation. Paradoxically, it also has nearly half of its population living below the poverty line, with 45.9% of people experiencing the challenge of having only one full meal every three days. A significant portion of the population resides in substandard conditions in areas prone to flooding, locally known as “hangover areas”. These conditions exacerbate issues such as violence, suicide rates, and public health concerns, while leaving 20.2% of the labor force unemployed. Consequently, all these factors significantly contribute to the high prevalence of TB within the community due to TB. It is crucial to develop strategies that not only focus on prevention, diagnosis, treatment, and recovery/rehabilitation, but also prioritize sustainable and environmentally balanced solutions for the Amazon region.

It is known that TB is a multifactorial disease influenced by various factors, ranging from bacillus infection to the manifestation of symptoms. These factors include socioeconomic and genetic characteristics, which can both positively and negatively influence the disease prognosis [43]. Coinfection with HIV, the social stigma associated with the disease that can hinder prompt healthcare seeking, difficulties in accessing healthcare services, and its close relationship with poverty and social vulnerability are noteworthy factors that influence the onset and prognosis of TB [36].

It is important to mention that although TB is a preventable and curable disease, the majority of cases still affect populations that are difficult to reach and living in situations of social vulnerability, especially in metropolitan areas and capitals of poor or developing countries. This can be explained by rapid population growth, which has intensified the process of social stratification and, consequently, the formation of more vulnerable population groups not only to TB but also to other infectious diseases [44].

Therefore, implementing policies that prioritize sustainability is of utmost importance for improving living conditions for the people residing in the region. Throughout history, the prevalence of poverty in the area has become evident, stemming from the decolonization process and the establishment of a dependent labor market characterized by extremely precarious conditions. It is crucial to develop new strategies and effective public policies that address the principles of social justice, taking into account the unique characteristics of the Amazon region. Merely implementing compensatory policies like *Bolsa Família*, aimed at reducing social inequalities, is not sufficient [45].

The study contributes to advancing knowledge by providing evidence of the structural and intermediary social determinants of TB in Eastern Amazonia. However, the model used in the study only considered TB cases. It would be beneficial to further explore other aspects of the disease, such as infection, using mathematical models to determine if they align with the findings related to the disease [4]. We utilized GAMLSS, which highlighted significant aspects for comprehending the TB context and the impact of structural and intermediary determinants on communities affected by TB. This allowed us to estimate the number of cases for each territory or UCT under investigation.

Although most studies have applied the frequentist/deterministic model to predict tuberculosis, this is the first that used the GAMLSS and evidenced important results to corroborate the End TB strategy and health policy. In terms of future works, it would be interesting to carry out a qualitative study specifically in areas where this study evidenced more problems with TB, using the ethnographic, anthropological and phenomenological studies to evidence what are the main barriers of this population to seek care. In addition, studies with health managers, health professionals as well as with the traditional healers through mixed methods would be strategic to understand the TB situation in those scenarios.

### Limitations

Logistic regression (or even linear regression) is a data analysis technique that uses mathematics to find the relationships between two data factors, therefore it can also be considered as a mathematical model, which can be used both to identify variables associated with the outcome of interest and to make predictions. However, there is a range of mathematical distributions that can better fit the data and be used to build more robust, accurate and reliable mathematical models. Thus, in the present study, we chose to use the GAMLSS technique to choose the best distribution and thereby elaborate a good model.

We used a more usual approach, which did not assume the existence of the constraint, which may be a potential limitation of the study. This is the first study applied in Eastern Amazonia that confirms its novelty and originality. Unfortunately, Brazil has not carried out its Demographic Census yet, mainly because of the budget shortfall. Therefore, the social situation identified may have worsened, mainly due to the COVID-19 pandemic, which means that the goal to End TB by 2050 seems more distant than we think.

## Conclusions

This study described the effects of the measure of association between structural and intermediate social determinants and tuberculosis in the Eastern Amazon region of Brazil. It represents a novel investigation into the social determinants associated with TB in an endemic area of the Amazon.

It is noteworthy that this is the first study conducted in the region. Based on the mathematical model, we identified an association between territories with the highest number of tuberculosis cases and those with deficient or absent sewage disposal systems. Additionally, there was a higher prevalence of tuberculosis among younger individuals (16 to 30 years old) and older individuals (above 61 years old). Furthermore, access to basic sanitation was found to create a favorable context for TB.

The present study highlights how the main social determinants of health are interconnected with each other and with the structure of societies through various social interactions, norms, and institutions that can impact population health. In light of the aforementioned, tuberculosis should be regarded as a process that primarily affects individuals who are part of specific social organizations. Therefore, advancing in the control and elimination of the disease is impossible without integrating government sectors to reduce poverty, inequality, and social exclusion, as well as improving access to quality healthcare services.

## Supplementary Information

**Additional file 1.**

**Additional file 2.**

## Data Availability

The datasets used and/or analysed during the current study are available from the corresponding author on reasonable request.

## Abbreviations

TB: Tuberculosis
WHO: World Health Organization
UN: United Nations
SDGs: Sustainable Development Goals
CDSS: Commission on Social Determinants of Health
PHC: Primary Health Care
GAMLSS: Generalized additive models for location, scale, and shape
ICD: International Classification of Diseases
SINAN: Notifiable Diseases Information System
HIV: Human Immunodeficiency Virus
AFB: Acid-Fast Bacilli
UCT: Urban Census Tract
IBGE: Brazilian Institute of Geography and Statistics
GAIC: Generalized Akaike Information Criterion
AIC: Akaike Information Criterion
VIF: Variance Inflation Factor
DPO: Double Poisson distribution
Q-Q plot: Quantile-Quantile plot
LRT: Likelihood Ratio Test
ZINBI: Zero Inflated Negative Binomial Distribution
ZANBI: Zero adjusted negative binomial distribution
ZABNB: Zero adjusted (hurdle) of the beta negative binomial distribution
ZIBNB: Zero-inflated of the beta negative binomial distribution
ZINBF: Zipf and zero adjusted Zipf distributions
ZISICHEL: Sichel distribution
ZIPIG: Zero-inflated Poisson inverse Gaussian distribution
SNBII: Negative Binomial type II distribution
SBNBI: Negative Binomial type I distribution
COVID-19: Corona Virus Disease 2019
